# Multi-omics analysis reveals novel loci and a candidate regulatory gene of unsaturated fatty acids in soybean (*Glycine max* (L.) Merr)

**DOI:** 10.1186/s13068-024-02489-2

**Published:** 2024-03-16

**Authors:** Xunchao Zhao, Yuhang Zhan, Kaiming Li, Yan Zhang, Changjun Zhou, Ming Yuan, Miao Liu, Yongguang Li, Peng Zuo, Yingpeng Han, Xue Zhao

**Affiliations:** 1 Key Laboratory of Soybean Biology in Chinese Ministry of Education (Key Laboratory of Soybean Biology and Breeding/Genetics of Chinese Agriculture Ministry), Northeast Agricultural University, Harbin, 150030 China; 2 Daqing Branch, Heilongjiang Academy of Agricultural Science, Daqing, China; 3 Qiqihar Branch, Heilongjiang Academy of Agricultural Science, Qiqihar, China; 4 Crop Tillage and Cultivation Institute, Heilongjiang Academy of Agricultural Sciences, Harbin, China

**Keywords:** Soybean, 3VmrMLM, Unsaturated fatty acids, Multi-omics analysis, Expression profile

## Abstract

**Background:**

Soybean is a major oil crop; the nutritional components of soybean oil are mainly controlled by unsaturated fatty acids (FA). Unsaturated FAs mainly include oleic acid (OA, 18:1), linoleic acid (LLA, 18:2), and linolenic acid (LNA, 18:3). The genetic architecture of unsaturated FAs in soybean seeds has not been fully elucidated, although many independent studies have been conducted. A 3 V multi-locus random single nucleotide polymorphism (SNP)-effect mixed linear model (3VmrMLM) was established to identify quantitative trait loci (QTLs) and QTL-by-environment interactions (QEIs) for complex traits.

**Results:**

In this study, 194 soybean accessions with 36,981 SNPs were calculated using the 3VmrMLM model. As a result, 94 quantitative trait nucleotides (QTNs) and 19 QEIs were detected using single-environment (QTN) and multi-environment (QEI) methods. Three significant QEIs, namely rs4633292, rs39216169, and rs14264702, overlapped with a significant single-environment QTN.

**Conclusions:**

For QTNs and QEIs, further haplotype analysis of candidate genes revealed that the *Glyma.03G040400* and *Glyma.17G236700* genes were beneficial haplotypes that may be associated with unsaturated FAs. This result provides ideas for the identification of soybean lipid-related genes and provides insights for breeding high oil soybean varieties in the future.

**Supplementary Information:**

The online version contains supplementary material available at 10.1186/s13068-024-02489-2.

## Background

Soybean [*Glycine max* (L.) Merr.], a major oil crop, is commonly used in cooking oil [1]. Soybean oil is mainly composed of saturated fatty acids (FAs) and unsaturated FAs. Among them, saturated FAs include palmitic and stearic acids, and unsaturated FAs include oleic (OA), linoleic acid (LLA), and linolenic acids (LNA) [2, 3]. Unsaturated FA is the main component of vegetable oil, accounting for more than 80% [4]. The increase in the content of OA, a monounsaturated FA, can improve oxidative stability and prevent oxidation [4]. LLA and LNA are polyunsaturated FAs and are very beneficial to human health [5]. However, the LLA and LNA show poor stability at a high temperature and are easily oxidized [6]. Thus, an important goal of soybean breeders is to increase the OA level and reduce the LLA and LNA content [7, 8].

Genome-wide association study (GWAS) mapping can identify the genetic basis of a variety of complex traits [9]. To date, the single-locus GWAS method has been widely applied to mine genetic loci underlying important agronomic traits, including 100-seed weight in soybean and oil content and yield-related traits in maize [2, 10, 11]. However, quantitative trait nucleotides (QTNs) have been detected using the single-locus GWAS method, which has limited ability to detect QTNs because quantitative traits are affected by a polygenic background [12].

Currently, the mixed linear model (MLM) method can correct population structure and family relationships and is widely used [13]. Based on the MLM method, single-locus GWAS methods have been widely proposed, including EMMAX, FaST-LMM and GEMMA [12, 14, 15]. However, single-locus GWAS methods generally require Bonferroni correction and can be affected by a polygenic background. To overcome this problem in single-locus GWAS methods, multi-locus GWAS methods have been applied, in which statistics are applied to all loci [16]. These multi-locus GWAS methods mainly include FASTmrEMMA, FASTmrMLM, FarmCPU, and pLARmEB [17–20]. However, these methods have a high calculation burden, and the advantages of QTN-by-environment interactions (QEIs) have not been fully considered.

To address this, a new multi-locus GWAS model, the 3 V multi-locus random-SNP-effect mixed linear model (3VmrMLM), has been presented [21]. This method improves the QTL detection capability and can analyze the genetic variation of complex traits. It provides a new method for the gene discovery of complex traits.

In this study, a dataset of 194 soybean accessions with 36,981 SNPs was applied [2]. We analyzed the unsaturated FA content in this population of 194 soybean accessions based on the multi-locus GWAS model (3VmrMLM). Our aim was to detect significant QEIs and stable QTNs compared with the results of our previous study and other independent studies and to further identify candidate genes related to unsaturated FA content.

## Results

### Phenotypic variation of three soybean unsaturated FA compositions

The distribution of unsaturated FA content (including OA, LLA, and LNA) in the 194 soybean accessions is shown in Table 1. The coefficient of variation (CV%) differed among the three years. In 2013, the unsaturated FA content had the highest CV at 51% (OA), 48% (LLA), and 52% (LNA). In 2014, the CVs of OA, LLA, and LNA were relatively consistent at 28%, 25%, and 29%, respectively. In 2015, the CV of unsaturated FAs was basically the same as in 2014 (Table 1). The heritabilities of OA, LLA, and LNA were 0.41, 0.36, and 0.35, respectively (Table 1). The above results showed that the content of unsaturated FAs was affected by the environment.

**Table 1:** Statistical analysis of oleic, linoleic, and linolenic acid traits

Trait	Years	Min	Max	Mean	SD	CV (%)	Heritability
Oleic acid content	2013	10.96	29.91	17.46	8.89	51	0.41
	2014	15.26	31.51	20.09	5.73	28	
	2015	14.86	34.81	18.19	4.9	26	
Linoleic acid content	2013	47.08	63.29	44.52	21.76	48	0.36
	2014	47.36	58.6	50.89	13.21	25	
	2015	44.06	60.68	53.35	12.59	23	
Linolenic acid content	2013	4.4	12.91	6.38	3.34	52	0.35
	2014	4.44	11.46	7.62	2.22	29	
	2015	4.23	13.34	8.72	2.29	26	

**Fig. 1:**
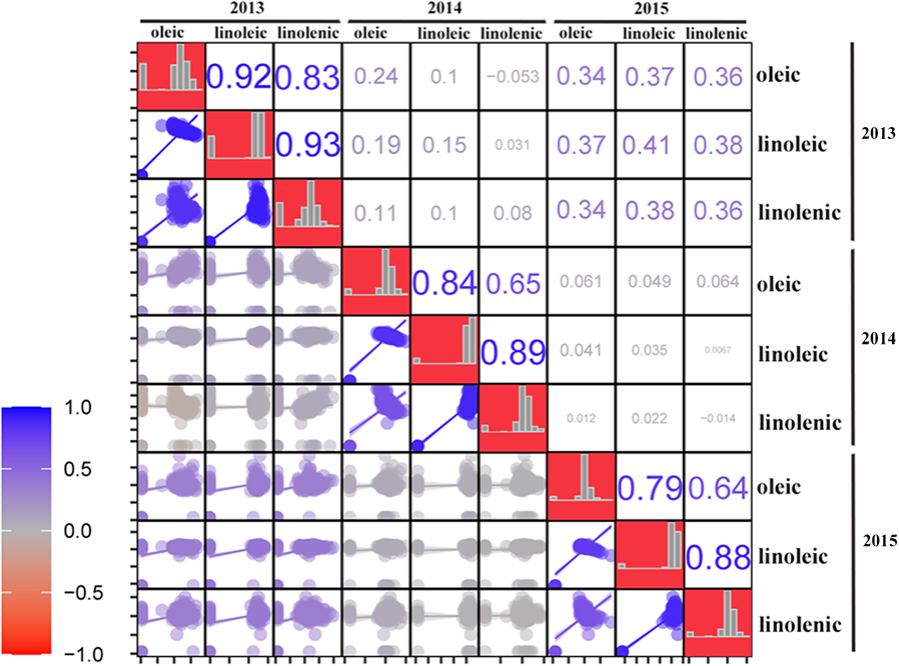
Distribution of oleic, linoleic, and linolenic traits in soybean and Pearson coefficients

The correlation coefficient of the unsaturated FA content was calculated. As shown in Fig. 1, OA, LLA, and LNA content had a high correlation within the same year. However, the OA, LLA, and LNA content was not high between different years. In 2013, OA was positively correlated with LLA and LNA (0.92 and 0.83, respectively). In 2014, OA was positively correlated with LLA and LNA (0.84 and 0.65, respectively). In 2015, OA was positively correlated with LLA and LNA (0.79 and 0.64, respectively). These results show that unsaturated FAs affect soybean oil accumulation.

### Identification of QTNs for unsaturated FA-related traits using 3VmrMLM

In this study, the unsaturated FA content was reanalyzed using the single-environment QTN model (3VmrMLM). A total of 94 significant QTNs were associated with the unsaturated FA content (LOD score ≥ 3.0). Among them, 30, 34, and 30 QTNs were associated with OA, LLA, and LNA content, respectively (Table 2).

**Table 2:** QTNs identified for unsaturated fatty acid content using the QTN detection model in 3VmrMLM

Trait	Year	SNP	Chr	Position (bp)	LOD	Additive effect	Dominant effect	Variance	*r*^2^ (%)	*p*-value	Known QTL	References
Oleic	2013	rs25094202	2	25094202	25.92	− 1.53	− 0.54	0.35	3.88	1.2E−26		
		rs6124789	4	6124789	19.04	− 1.22	− 0.43	0.31	3.45	9E−20		
		rs40652518	4	40652518	19.99	− 1.23		0.76	8.48	8.5E−22		
		rs14903097	6	14903097	6.23	− 0.39	− 1.78	0.14	1.59	5.9E−07		
		rs29444284	6	29444284	11.36	0.68	− 2.62	0.58	6.50	4.4E−12		
		rs32503987	6	32503987	17.07	− 1.11		0.57	6.41	7.6E−19		
		rs545702	7	545702	28.94	1.61	1.24	0.84	9.37	1.2E−29		
		rs29716728	7	29716728	3.32	0.44	− 0.06	0.17	1.96	0.00048		
		rs19742379	8	19742379	11.13	0.01	7.58	0.72	8.10	7.5E−12		
		rs2762447	9	2762447	13.18	− 0.17	− 3.02	0.70	7.87	6.6E−14		
		rs18612544	9	18612544	4.55	0.04	3.75	0.26	2.95	2.8E−05		
		rs15583833	11	15583833	6.67	0.64		0.28	3.12	3E−08		
	2014	rs25962449	3	25962449	13.08	− 0.98	− 0.06	0.21	3.10	8.3E−14		
		rs9480030	4	9480030	23.29	1.37		0.74	10.93	3.9E−25		
		rs24966112	4	24966112	10.53	− 0.91	0.26	0.22	3.23	2.9E−11		
		rs37372282	7	37372282	6.50	− 0.65		0.27	3.95	4.5E−08		
		rs13246407	8	13246407	12.51	− 0.79	4.87	0.45	6.75	3.1E−13		
		rs4633292	9	4633292	6.26	0.63	0.60	0.36	5.41	5.5E−07		
		rs7608184	11	7608184	7.75	− 0.69	2.38	0.33	4.96	1.8E−08		
		rs86980	14	86980	6.96	0.67		0.36	5.27	1.5E−08		
		rs21488603	16	21488603	7.07	− 0.57	1.80	0.35	5.25	8.6E−08		
		rs42357728	19	42357728	9.79	0.78	2.22	0.23	3.48	1.6E−10		
	2015	rs14131618	2	14131618	9.20	0.62	5.83	0.37	5.75	6.3E−10		
		rs648720	3	648720	7.62	− 0.21	7.18	0.59	9.19	2.4E−08		
		rs1225280	12	1225280	4.41	− 0.58	− 0.73	0.32	4.92	3.9E−05		
		rs16802809	13	16802809	8.65	− 0.83		0.30	4.73	2.8E−10		
		rs19292381	13	19292381	9.58	− 0.47	5.11	0.70	10.91	2.7E−10		
		rs30304375	13	30304375	3.38	0.07	3.91	0.25	3.81	0.00042		
		rs23631249	17	23631249	6.54	0.72		0.45	6.94	4.1E−08	Seed oleic 6–9	[22]
		rs37263037	17	37263037	7.38	0.74	− 1.42	0.28	4.36	4.2E−08	Seed oleic 6–9	[22]
Linoleic	2013	rs39587481	2	39587481	16.48	− 0.83	− 0.77	0.15	1.93	3.3E−17		
		rs28569907	3	28569907	24.14	− 1.09	− 0.06	0.26	3.36	7.3E−25		
		rs41402037	4	41402037	12.32	0.70	0.45	0.37	4.68	4.8E−13		
		rs48166843	4	48166843	11.78	− 0.68	0.44	0.17	2.22	1.7E−12		
		rs4602961	9	4602961	12.39	0.69	− 1.08	0.45	5.71	4.1E−13		
		rs11516441	9	11516441	15.27	− 0.80		0.37	4.75	5E−17		
		rs38142875	9	38142875	12.93	0.54	− 4.23	0.31	3.99	1.2E−13		
		rs9167160	10	9167160	8.83	0.58		0.32	4.05	1.8E−10		
		rs46493899	10	46493899	7.06	0.47	− 1.85	0.25	3.26	8.8E−08	Seed oleic 6–10	[22]
		rs34135970	12	34135970	11.07	− 0.36	2.82	0.42	5.43	8.6E−12		
		rs1501986	14	1501986	6.23	0.47		0.20	2.59	8.6E−08		
		rs48109369	14	48109369	5.18	− 0.07	2.65	0.18	2.35	6.7E−06		
		rs6755025	15	6755025	5.60	− 0.44	− 0.98	0.17	2.16	2.5E−06		
		rs32005810	16	32005810	4.43	0.39	− 0.66	0.14	1.80	3.7E−05		
		rs30412910	17	30412910	11.47	0.64	− 2.71	0.40	5.17	3.4E−12		
		rs9070866	18	9070866	6.15	0.28	− 3.37	0.18	2.30	7.1E−07		
		rs4876597	19	4876597	7.75	0.22	1.86	0.18	2.28	1.8E−08		
	2014	rs4953186	2	4953186	20.77	− 1.01	0.66	0.24	6.07	1.7E−21	rs4953186	[2]
		rs14587513	2	14587513	5.80	0.47		0.22	5.71	2.4E−07		
		rs6481810	3	6481810	17.03	0.86	2.05	0.36	9.28	9.4E−18	rs6481810	[2]
		rs24966112	4	24966112	18.84	1.00	− 0.08	0.20	5.21	1.5E−19		
		rs17451809	6	17451809	3.43	− 0.36		0.12	3.16	7.1E−05		
		rs14264702	7	14264702	14.81	− 0.80		0.20	5.28	1.5E−16		
		rs13246407	8	13246407	4.89	0.28	− 3.14	0.14	3.59	1.3E−05		
		rs37217687	12	37217687	3.61	− 0.22	− 2.31	0.12	2.99	0.00025		
		rs9446804	15	9446804	6.72	− 0.12	− 2.23	0.22	5.54	1.9E−07	Seed oleic 4–1	[36]
		rs39216169	17	39216169	5.23	− 0.45		0.12	3.11	9.2E−07		
	2015	rs25887810	1	25887810	17.08	1.04		0.26	6.29	7.3E−19		
		rs46294440	6	46294440	4.98	− 0.51	− 0.85	0.27	6.39	1E−05		
		rs36974795	12	36974795	7.52	0.46	− 2.09	0.33	7.85	3E−08		
		rs16802809	13	16802809	7.43	0.64		0.18	4.33	4.9E−09		
		rs25691662	13	25691662	8.76	− 0.27	− 6.21	0.39	9.48	1.7E−09		
		rs21353920	15	21353920	17.96	1.07	0.40	0.28	6.83	1.1E−18		
		rs14234658	18	14234658	6.51	− 0.18	− 3.45	0.29	6.96	3.1E−07		
Linolenic	2013	rs7344385	2	7344385	9.69	0.44		0.13	6.80	2.4E−11		
		rs5009451	3	5009451	7.39	− 0.38		0.09	4.47	5.4E−09		
		rs15602593	4	15602593	18.65	0.64	− 0.80	0.14	6.99	2.2E−19		
		rs35874955	4	35874955	4.18	− 0.13	1.36	0.07	3.69	6.6E−05		
		rs45800101	14	45800101	6.16	− 0.34	0.01	0.08	4.13	6.9E−07		
		rs30575841	15	30575841	7.74	0.13	1.86	0.12	5.96	1.8E−08		
		rs4718078	17	4718078	22.54	− 0.74		0.15	7.90	2.3E−24		
		rs41437124	17	41437124	4.82	0.06	2.10	0.08	4.14	1.5E−05		
		rs47611415	18	47611415	7.11	0.29	− 1.06	0.10	5.10	7.7E−08		
		rs18173239	20	18173239	7.13	0.37	− 0.68	0.05	2.39	7.5E−08		
	2014	rs42280985	4	42280985	7.76	0.03	1.43	0.08	6.95	1.8E−08		
		rs42904836	4	42904836	9.14	0.33	0.27	0.03	2.59	7.3E−10		
		rs4633296	9	4633296	10.72	− 0.36	0.83	0.12	10.00	1.9E−11		
		rs21698230	14	21698230	5.68	− 0.25		0.04	3.36	3.2E−07	Seed oleic 7–3	[25]
		rs5443771	16	5443771	5.07	− 0.24	0.31	0.06	4.72	8.5E−06		
		rs39098643	17	39098643	6.31	− 0.28	− 0.15	0.06	5.16	4.9E−07		
		rs41268049	17	41268049	5.73	0.10	− 1.02	0.06	5.41	1.8E−06		
		rs52833743	18	52833743	10.50	− 0.36		0.06	5.04	3.5E−12	rs52833743; Seed oleic 3–3; Seed oleic 5–2	[2, 24]
	2015	rs6099191	2	6099191	8.39	0.14	2.49	0.06	5.41	4.1E−09		
		rs5660143	3	5660143	9.24	0.12	2.23	0.08	6.82	5.8E− 10		
		rs32069131	6	32069131	20.28	0.51	− 0.14	0.06	4.86	5.3E−21		
		rs38597109	8	38597109	18.99	− 0.47		0.07	5.93	8.6E−21		
		rs18435729	10	18435729	6.46	− 0.01	2.43	0.06	5.37	3.5E−07		
		rs39609078	12	39609078	6.66	− 0.26		0.05	4.29	3.1E−08		
		rs2599191	14	2599191	10.61	0.33	− 0.06	0.09	7.78	2.5E−11		
		rs9834940	14	9834940	9.71	0.32	− 0.21	0.04	3.58	2E−10		
		rs26951252	14	26951252	10.54	0.30	− 0.66	0.08	6.90	2.9E−11	Seed oleic 6–10; Seed oleic 7–3	[22, 25]
		rs21822969	15	21822969	7.38	0.05	2.56	0.07	5.98	4.2E−08		
		rs30575841	15	30575841	15.05	− 0.40	0.58	0.07	5.56	9E−16		
		rs35024325	17	35024325	12.77	0.37	− 0.18	0.03	2.78	1.7E−13	rs35024325; Seed oleic 6–9	[2, 22]

In 2013, 2014, and 2015, 12, 10, and eight QTNs were associated with OA content, with LOD scores of 4.55–25.92, 6.25–23.29, and 3.37–9.57, respectively. A total of 17, 10, and seven QTNs associated with LLA content were identified with LOD scores of 4.43–24.14, 3.42–20.76, and 4.98–17.08 in 2013, 2014, and 2015, respectively. In 3 years (2013, 2014, and 2015), 10, eight, and 12 QTNs associated with LNA content were detected with LOD scores of 4.17–22.53, 5.07–10.72, and 6.46–20.27, respectively (Table 2, Additional file 1: Fig. S1).

### Detection of QEIs for unsaturated FA content using 3VmrMLM with multiple environments

**Fig. 2:**
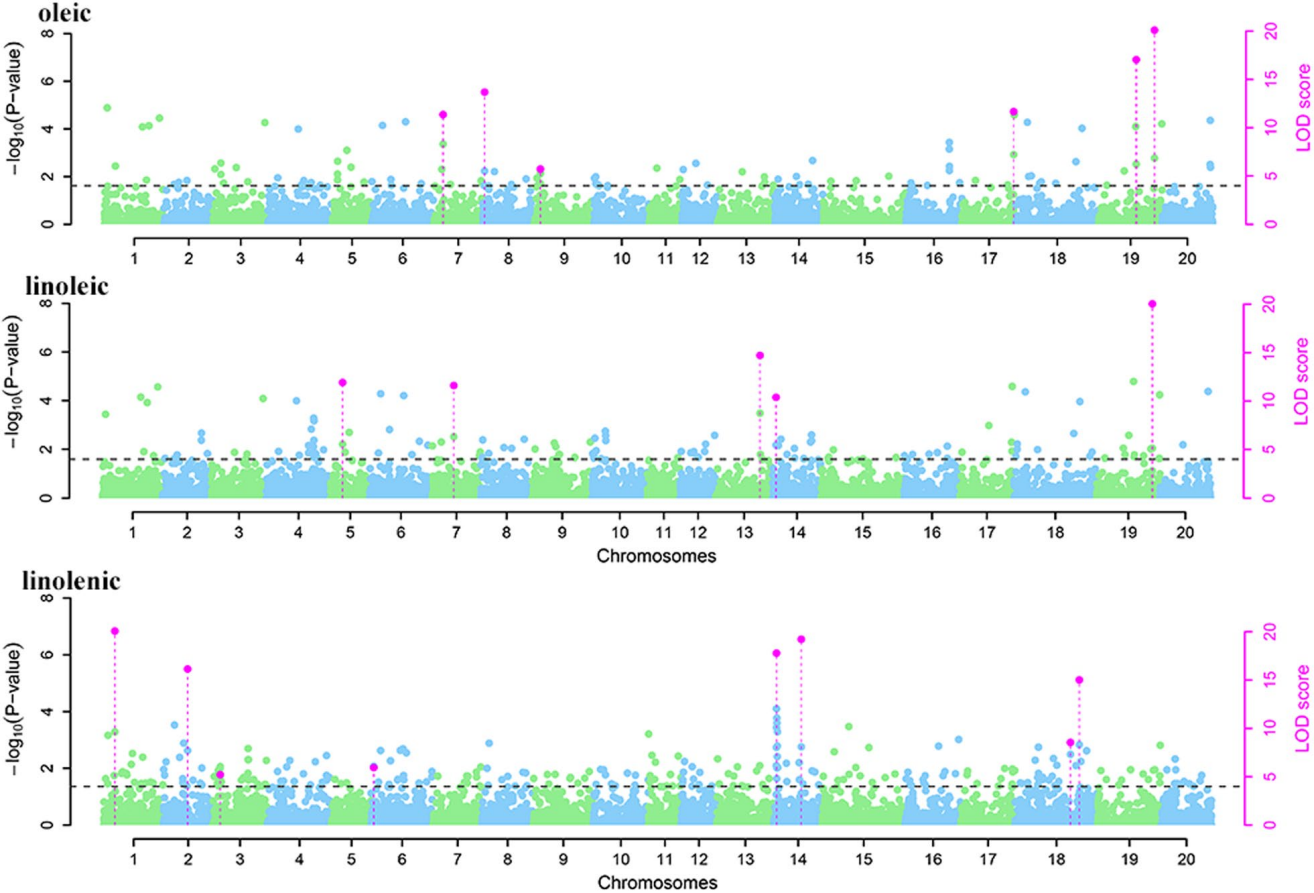
Manhattan plots of the multi-environment analysis for the oleic, linoleic, and linolenic acid content in soybean seeds

The unsaturated FA content was reanalyzed in 3 years (2013, 2014, and 2015) using the multiple-environment QEI model (3VmrMLM) for identifying QEIs. A total of 19 significant/suggested QEIs were identified (Table 3, Fig. 2). Three significant QEIs overlapped with the above QTNs. In these QEIs, the r^2^ value was between 2.01 and 14.67, and the variance value was between 0.03 and 1.33 (Table 3).

**Table 3:** Significant/suggested QEIs for soybean unsaturated fatty acid content in three environments detected using the QTN-by-environment detection model in 3VmrMLM

Trait	SNP	Chr	Position (bp)	LOD (QE)	add* env1	dom* env1	add* env2	dom* env2	add* env3	dom* env3	variance	*r*^2^ (%)	*p*-value	**Known QTL**	**References**
Oleic	rs6528670	7	6528670	11.36	0.58	− 4.19	0.08	0.21	− 0.66	3.97	0.43	4.71	1.10E−10		
Oleic	rs1902760	8	1902760	13.68	0.66	4.37	− 0.45	0.46	− 0.22	− 4.83	0.55	6.09	6.70E−13		
Oleic	rs4633292	9	4633292	5.72	0.01	0.19	0.53	− 1.73	− 0.54	1.55	0.21	2.31	2.60E−05		
Oleic	rs39216169	17	39216169	11.67	0.20		0.69		− 0.89		0.44	4.82	2.10E−12		
Oleic	rs31881460	19	31881460	17.04	− 0.52	− 0.57	− 0.63	− 0.33	1.15	0.91	0.66	7.32	3.60E−16		
Oleic	rs44492166	19	44492166	30.20	− 1.49		0.18		1.31		1.33	14.67	6.30E−31		
Linoleic	rs12727785	5	12727785	11.91	− 0.82	− 0.76	0.04	1.00	0.78	− 0.24	0.43	6.28	3.40E−11		
Linoleic	rs14264702	7	14264702	11.60	0.04		− 0.78		0.74		0.39	5.68	2.40E−12	Seed oleic 6–7	[22]
Linoleic	rs34595703	13	34595703	14.71	− 0.94	0.03	0.08	− 0.16	0.86	0.13	0.54	7.87	6.80E−14	Seed oleic 6–4	[22]
Linoleic	rs2457629	14	2457629	10.39	0.25		0.56		− 0.81		0.35	5.09	4.10E−11		
Linoleic	rs44492166	19	44492166	22.99	1.17		− 0.05		− 1.12		0.87	12.75	1.01E−23	Seed linoleic 2–2; Seed linolenic 2–3	[26]
Linolenic	rs8152225	1	8152225	26.67	0.39	0.39	0.23	0.12	− 0.61	− 0.51	0.19	10.79	1.30E−25		
Linolenic	rs23852645	2	23852645	16.14	− 0.10	0.98	− 0.33	− 1.08	0.43	0.11	0.11	6.04	2.70E−15	Seed linoleic 7–1	[25]
Linolenic	rs4406682	3	4406682	5.22	− 0.24		0.04		0.21		0.03	2.01	6.09E−06		
Linolenic	rs2988645	6	2988645	5.95	− 0.03	2.02	− 0.07	0.16	0.10	− 2.17	0.04	2.21	1.60E−05		
Linolenic	rs1701848	14	1701848	17.78	− 0.40		− 0.05		0.45		0.12	7.09	1.60E−18		
Linolenic	rs26951255	14	26951255	19.22	0.35	− 1.22	0.13	1.19	− 0.48	0.03	0.13	7.64	2.70E−18	Seed linolenic 10–1; Seed linolenic 7–6	[22]
Linolenic	rs42367957	18	42367957	8.55	− 0.09	− 1.31	0.00	− 0.79	0.10	2.10	0.05	3.06	5.80E−08		
Linolenic	rs48948953	18	48948953	15.02	0.11		− 0.43		0.32		0.10	5.60	9.60E−16

### Candidate gene prediction of significant QTNs associated with unsaturated FA in soybean

There were 1246 genes identified in the flanking genomic region of each significant QTN using the 3VmrMLM method (Additional file 1: Table S1). We further conducted the Kyoto Encyclopedia of Genes and Genomes (KEGG) analysis. As shown in Additional file 1: Fig. S2A, 201 genes were significantly enriched in metabolism, genetic information processing, environmental information processing, cellular processes, and organismal systems, including lipid metabolism, amino acid metabolism, energy metabolism, transport, and catabolism. The results of the above enrichment analysis showed that some candidate genes around QTN were found in different processes.

The same methods mentioned above were used to analyze candidate genes in the flanking regions of the QEIs. A total of 301 candidate genes were found in the linked regions of significant QEIs (Additional file 1: Table S2). KEGG analysis found that 53 genes were significantly enriched in metabolism, genetic information processing, environmental information processing, and organismal systems, including carbohydrate metabolism and lipid metabolism (Additional file 1: Fig. S2B). In the multiple-environment QEI model, five known SNP markers were identified. In addition, some new SNP markers, including rs6528670, rs1902760, rs4633292, rs2457629, and rs48948953, were related to FA synthesis. Moreover, some known markers were identified in the multiple-environment QEI model, including rs14264702, rs34595703, rs44492166, rs23852645, and rs26951255. Three significant QEIs, namely rs4633292, rs39216169, and rs14264702, overlapped with significant QTN in a single year, of which rs14264702 has been reported [22].

### Transcriptomic analysis of HUFA and LUFA soybean seeds

RNA-seq analysis was conducted to reveal the transcriptional regulation of unsaturated FA metabolism in HUFA (high unsaturated fatty acid) and LUFA (low unsaturated fatty acid) soybean seeds. Three comparison groups were analyzed: a comparison group of five HUFA and five LUFA varieties (FHUFA vs. FLUFA); a comparison of 10 HUFA and 10 LUFA varieties (THUFA vs. TLUFA); and a comparison of 15 HUFA and 15 HUFA varieties (HUFA vs. LUFA) (Additional file 1: Table S3).

**Fig. 3:**
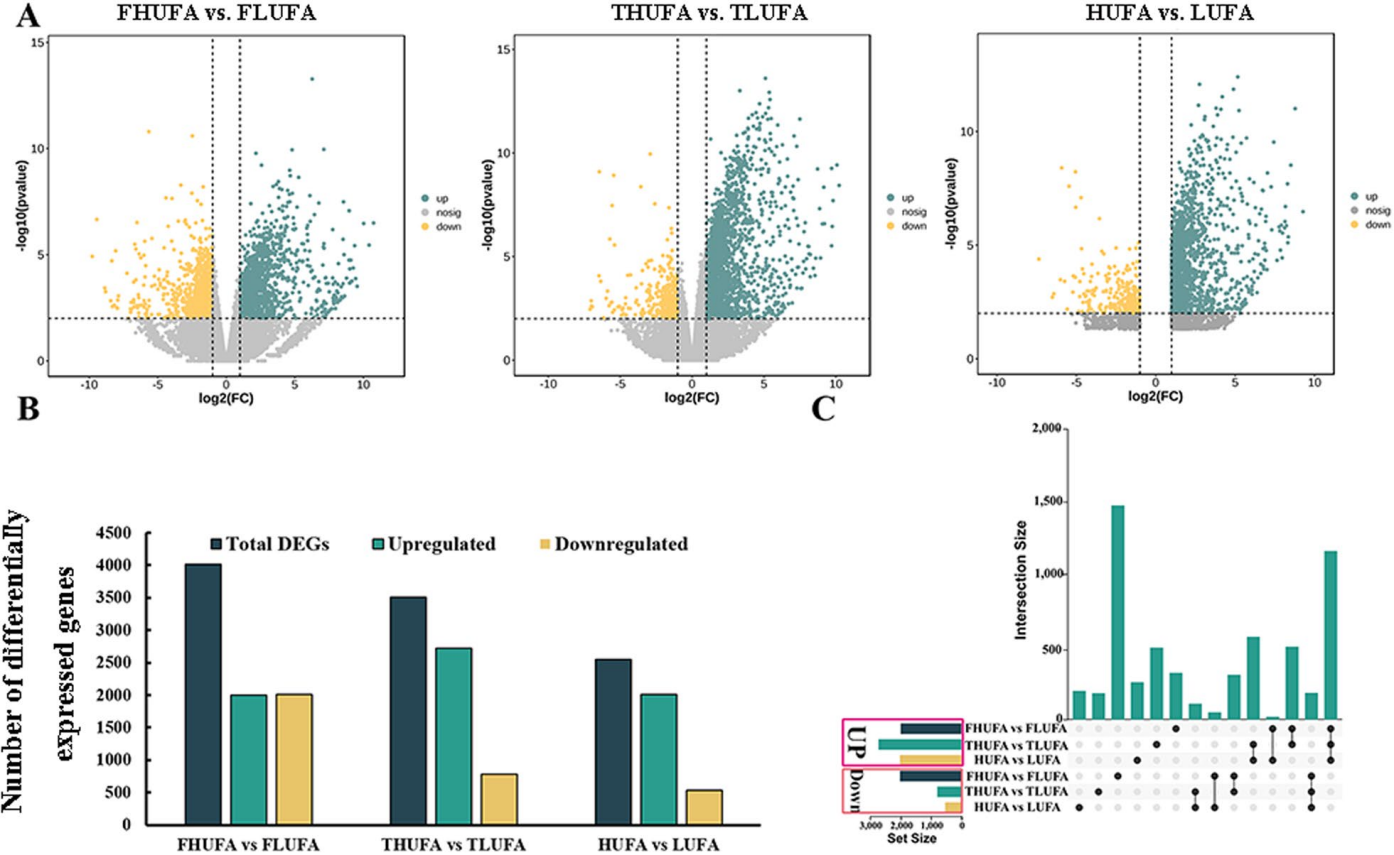
Multivariate statistical analysis of the transcriptome data in the soybean samples. **A** Volcano maps in different comparison groups. **B** Number of differentially expressed genes. The green and yellow columns represent the numbers of genes with upregulated and downregulated expression, respectively. **C** Upsetplot diagram showing the overlapping DEGs in the three comparison groups

There were 4013, 3504, and 2546 DEGs in the FHUFA vs. FLUFA, THUFA vs. TLUFA, and HUFA vs. LUFA groups, respectively (Fig. 3A, B). In each comparison group, the number of upregulated DEGs was higher than the number of downregulated DEGs. As shown in Fig. 3C, 1160 common DEGs were upregulated, while 183 common DEGs were downregulated.

### Identification of candidate genes by integrating GWAS and RNA-seq analysis

To further identify candidate genes, DEGs were identified by integrating GWAS and RNA-seq analysis and by analyzing potential candidate genes. In the single-environment QTN model, 91, 85, and 61 DEGs were found in the FHUFA vs. FLUFA, THUFA vs. TLUFA, and HUFA vs. LUFA groups, respectively (Additional file 1: Table S1). A total of 30 DEGs were found in all three comparison groups. Among them, *Glyma.10G079500*, *Glyma.19G163600*, *Glyma.09G033600*, and *Glyma.02G068900* genes were upregulated (Log2FC > 3), and *Glyma.07G205400*, *Glyma.09G053700*, and *Glyma.06G175100* genes were downregulated (Log2FC <  − 1) (Table 4, Additional file 1: Fig. S3A).

**Table 4:** Candidate genes are identified in the transcriptome and QTN detection model

Gene ID	Gene function	Arabidopsis homologs	FHUFA vs. FLUFA	THUFA vs. TLUFA	HUFA vs. LUFA
*Glyma.02G068900*	Xyloglucan endotransglucosylase/hydrolase 5	AT5G13870.1	4.86	6.48	3.93
*Glyma.02G210300*	Unknown protein	AT2G14095.1	2.84	3.01	2.75
*Glyma.03G006700*	cysteine synthase 26	AT3G03630.1	1.26	1.32	1.02
*Glyma.03G040000*	lipid transfer protein 2	AT2G38530.1	4.86	3.01	1.93
*Glyma.03G040100*	lipid transfer protein 1	AT2G38540.1	4.01	4.67	2.63
*Glyma.15G087200*	Aldolase-type TIM barrel family protein	AT5G01410.1	1.54	1.59	1.33
*Glyma.03G040500*	Unknown protein	AT2G40435.1	2.24	1.45	1.16
*Glyma.04G102400*	Unknown protein	AT1G78170.1	2.19	2.37	2.11
*Glyma.04G102700*	Major facilitator superfamily protein	AT1G34580.1	2.73	2.84	1.71
*Glyma.04G209200*	Amino acid permease 2	AT5G09220.1	1.05	2.19	1.61
*Glyma.06G175100*	Leucine-rich repeat protein kinase family protein	AT2G31880.1	− 1.51	− 1.26	− 1.11
*Glyma.07G205400*	Cysteine proteinases superfamily protein	AT3G49340.1	− 3.61	− 2.88	− 2.75
*Glyma.09G032100*	myb domain protein 78	AT5G49620.1	2.11	2.40	1.72
*Glyma.09G033500*	Unknown protein	AT5G49525.1	3.02	2.50	1.58
*Glyma.09G033600*	Unknown protein	no	9.01	5.17	5.18
*Glyma.09G051900*	VQ motif-containing protein	AT4G20000.1	2.70	3.95	3.23
*Glyma.17G236700*	Acyl-CoA-binding domain 3	AT4G24230.6	1.62	1.96	1.24
*Glyma.09G053700*	Ankyrin repeat family protein	AT3G54070.1	− 6.18	− 5.18	− 4.86
*Glyma.09G157500*	Unknown protein	no	1.94	2.03	1.39
*Glyma.10G079500*	Unknown protein	AT1G32120.1	8.32	6.32	6.43
*Glyma.11G100600*	Peptidoglycan-binding LysM domain-containing protein	AT5G23130.1	2.51	1.94	1.16
*Glyma.14G216100*	Protein kinase superfamily protein	AT5G37790.1	2.54	2.18	1.50
*Glyma.03G040400*	Lipid transfer protein 1	AT2G38540.1	1.26	1.94	1.42
*Glyma.15G088900*	GDSL-like lipase/acylhydrolase superfamily protein	AT1G29670.1	2.01	1.95	1.58
*Glyma.16G161500*	DNAse I-like superfamily protein	AT1G71710.1	1.61	1.77	1.20
*Glyma.17G220100*	Pentatricopeptide repeat (PPR) superfamily protein	AT2G13600.1	− 1.59	− 1.41	− 1.08
*Glyma.17G236200*	Salt tolerance zinc finger	AT1G27730.1	2.77	3.12	1.19
*Glyma.18G239700*	Wall-associated kinase-like 2	AT1G16130.1	3.43	3.05	3.28
*Glyma.18G239900*	Cytochrome P450, family 97, subfamily A, polypeptide 3	AT1G31800.1	1.67	1.49	1.11
*Glyma.19G163600*	RING/U-box superfamily protein	AT1G04360.1	5.88	7.34	5.37

In the multiple-environment QEI model, 26, 28, and 20 DEGs were found in the FHUFA vs. FLUFA, THUFA vs. TLUFA, and HUFA vs. LUFA groups, respectively (Additional file 1: Table S2). Among these candidate genes, nine were simultaneously detected by GWAS and common DEGs in all three comparison groups. These nine genes included those encoding an Acyl-CoA-binding protein (*Glyma.17G236700*), Ankyrin repeat family protein (*Glyma.09G053700*), Nodulin MtN3 family protein (*Glyma.08G025100*), Integrase-type DNA-binding superfamily protein (*Glyma.18G206600*), Calmodulin-domain protein kinase 9 (*Glyma.14G023500*), ARM repeat superfamily protein (*Glyma.03G036700*), Protein kinase superfamily protein (*Glyma.03G036000*), BRI1 kinase inhibitor 1 (*Glyma.06G039100*), NAC domain-containing protein 73 (*Glyma.13G234700*), and unknown function protein (*Glyma.18G205700*, *Glyma.18G205400*) (Table 5, Additional file 1: Fig. S3B). The expression of these genes was further determined by qRT-PCR and was basically consistent with that of the transcriptome data (Additional file 1: Fig. S4).

**Table 5:** Candidate genes are identified in the transcriptome and QEIs detection model

Gene ID	Gene function	Arabidopsis homologs	FHUFA vs FLUFA	THUFA vs TLUFA	HUFA vs LUFA
*Glyma.09G053700*	Ankyrin repeat family protein	AT3G54070.1	− 6.18	− 5.18	− 4.86
*Glyma.13G234700*	NAC domain containing protein 73	AT4G28500.1	− 1.56	− 1.14	− 1.08
*Glyma.18G206600*	Integrase-type DNA-binding superfamily protein	AT2G40340.1	1.21	1.85	1.24
*Glyma.17G236700*	Acyl-CoA-binding domain 3	AT4G24230.6	1.62	1.96	1.24
*Glyma.14G023500*	Calmodulin-domain protein kinase 9	AT3G20410.1	1.66	1.86	1.32
*Glyma.03G036000*	Protein kinase superfamily protein	AT5G01850.1	1.85	1.90	1.35
*Glyma.03G036700*	ARM repeat superfamily protein	AT5G01830.1	2.21	1.70	1.37
*Glyma.08G025100*	Nodulin MtN3 family protein	AT4G10850.1	2.43	1.67	1.24
*Glyma.18G205400*	Unknown protein	AT3G51750.1	2.43	4.03	6.30

### Metabolic profiling analysis of MHUFA and MLUFA soybean seeds

To determine the unsaturated FA regulatory network at the seed development stage, a non-targeted metabolic profiling analysis was applied. There were 15 high unsaturated FA (HUFA) and 15 low unsaturated FA (LUFA) soybean varieties applied in this study (Additional file 1: Table S3). Multiple metabolites were detected using non-targeted metabolomics, including secondary metabolites, lipids, amino acids, and flavonoids.

**Fig. 4:**
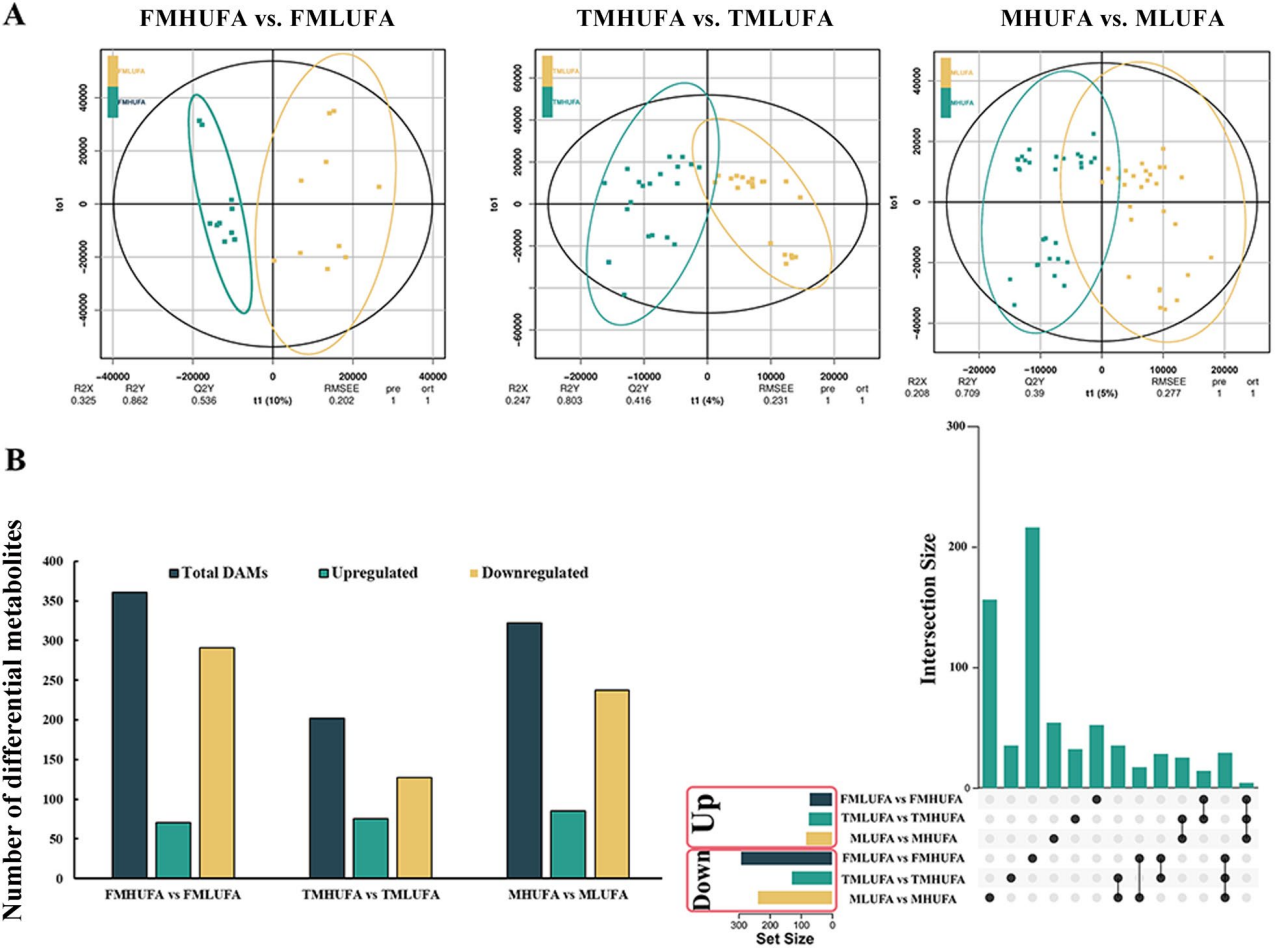
Multivariate statistical analysis of the metabolome data in the soybean samples. **A** OPLS-DA model analysis. **B** Number of differential metabolites. The green and yellow columns represent the number of genes that were upregulated and downregulated, respectively. **C** Upsetplot diagram showing the overlapping DAMs in the three comparison groups

To explore the differences in metabolites between different varieties, three comparison groups were studied: five high-unsaturated FA (FMHUFA) and five low-unsaturated FA (FMLUFA) varieties (FMHUFA vs. FMLUFA); 10 high-unsaturated FA (TMHUFA) and 10 low-unsaturated FA (TMLUFA) varieties (TMHUFA vs. TMLUFA); and 15 high-unsaturated FA (MHUFA) and 15 low-unsaturated FA (MLUFA) varieties (MHUFA vs. MLUFA). The OPLS-DA analysis showed that the model accurately described each sample and was suitable for subsequent analysis (Fig. 4A). According to the OPLS-DA model, 70 differentially abundant metabolites (DAMs) were upregulated, and 291 DAMs were downregulated in FMHUFA vs. FMLUFA (Fig. 4B). These metabolites included lipids, secondary metabolites, and unknown metabolites. In addition, 202 and 322 DAMs were identified in TMHUFA vs. TMLUFA and MHUFA vs. MLUFA, respectively (Fig. 4B). As shown in Fig. 4C, four common upregulated DAMs and 29 common downregulated DAMs were identified.

**Fig. 5:**
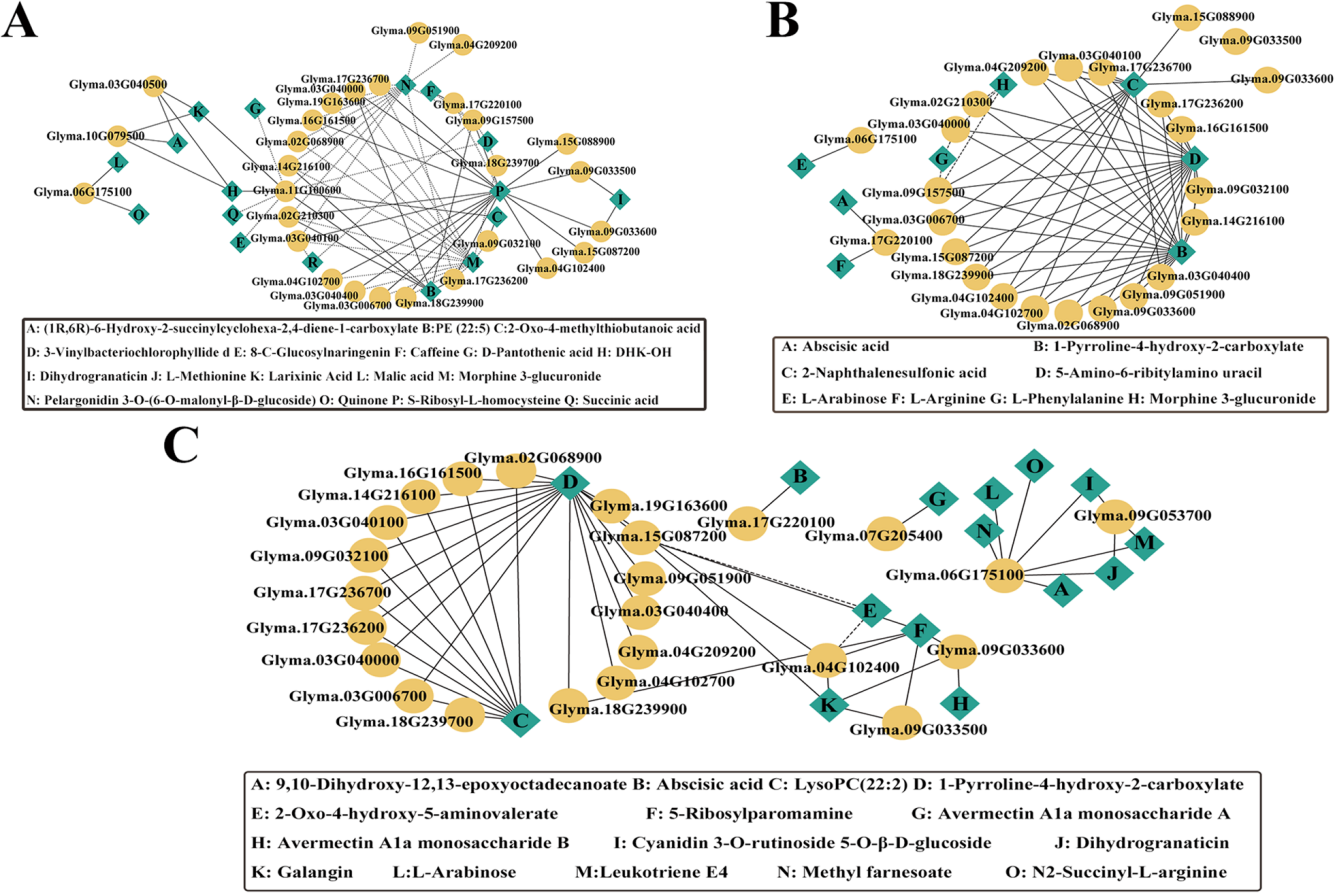
In the QTN detection model, network analysis of the candidate DEGs and DAMs in the three comparison groups. **A** FHUFA vs. FLUFA, **B** THUFA vs. TLUFA, and **C** HUFA vs. LUFA. Yellow circles represent genes. Green squares represent metabolites. The solid line represents a positive correlation, while the dashed line represents a negative correlation

### Differential accumulation of metabolites with MHUFA and MLUFA content

In this study, the metabolic changes of high and low unsaturated FA content in 30 soybean varieties during the R6 period were studied. In FMHUFA vs. FMLUFA, 29 DAMs were annotated into the KEGG pathway. Among them, the isoflavone pathway had the most DAMs, including Genistein, 8-C-glucosylnaringenin, genistin, and biochanin A (Additional file 1: Fig. S5A). In TMHUFA vs. TMLUFA, 16 DAMs were annotated into the KEGG pathway; among them, the TCA cycle had the most DAMs. 1-Pyrroline-4-hydroxy-2-carboxylate, 5-amino-6-ribitylamino uracil, and 2-(acetamidomethylene) succinate were differentially accumulated in TMHUFA vs. TMLUFA (Additional file 1: Fig. S5B). In MHUFA vs. MLUFA, 39 DAMs were annotated into the KEGG pathway, including the TCA cycle, LLA metabolism, and biosynthesis of amino acids. LysoPC (22:2(13Z,16Z)), (2S,5S)-trans-carboxymethylproline, and quercetin 3-sambubioside were differentially accumulated in MHUFA vs. MLUFA (Additional file 1: Fig. S5C).

### Co‑expression analysis of candidate genes and DAM metabolites

Candidate genes and metabolite networks were analyzed. In the single-environment model, the co-expression network of 30 candidate genes and DAMs in three comparison groups was constructed. In the FHUFA vs. FLUFA network, the results indicated that the 75 subnetworks were significantly correlated (|*r*|> 0.5,* p* < 0.05). PE (22:5) was positively associated with *Glyma.14G216100* (*r* > 0.51, *p* < 0.02), *Glyma.03G040000* (*r* > 0.51, *p* < 0.01), *Glyma.02G210300* (*r* > 0.51, *p* < 0.02), *Glyma.17G236200* (*r* > 0.52, *p* < 0.01), *Glyma.11G100600* (*r* > 0.59, *p* < 0.006), and *Glyma.09G157500* (*r* > 0.68, *p* < 0.0007). Quinone was positively associated with *Glyma.17G236700* (*r* > 0.53, *p* < 0.01) and *Glyma.03G040400* (*r* > 0.67, *p* < 0.001) (Fig. 5A). In the THUFA vs. TLUFA network, 1-pyrroline-4-hydroxy-2-carboxylate was positively associated with *Glyma.09G051900* (*r* > 0.88, *p* < 5.53E−14) and *Glyma.03040400* (*r* > 0.88, *p* < 2.35E−14). *Glyma.17G236700* was positively associated with 2-(acetamidomethylene) succinate (*r* > 0.51, *p* < 0.0007), 5-amino-6-ribitylamino uracil (*r* > 0.69, *p* < 6.85E−07), and 1-pyrroline-4-hydroxy-2-carboxylate (*r* > 0.69, *p* < 6.09E−07) (Fig. 5B). In the HUFA vs. LUFA network, 1-pyroline-4-hydroxy-2-carboxylate B was significantly associated with *Glyma.03G040400* (*r* > 0.84, *p* < 3.69E−17) and *Glyma.09G051900* (*r* > 0.85, *p* < 8.61E−18). *Glyma.17G236700* was significantly associated with LysoPC(22:2(13Z,16Z)) (*r* > 0.64, *p* < 3.04E−08) and 1-pyrroline-4-hydroxy-2-carboxylate B (*r* > 0.64, *p* < 2.01E−08) (Fig. 5C).

**Fig. 6:**
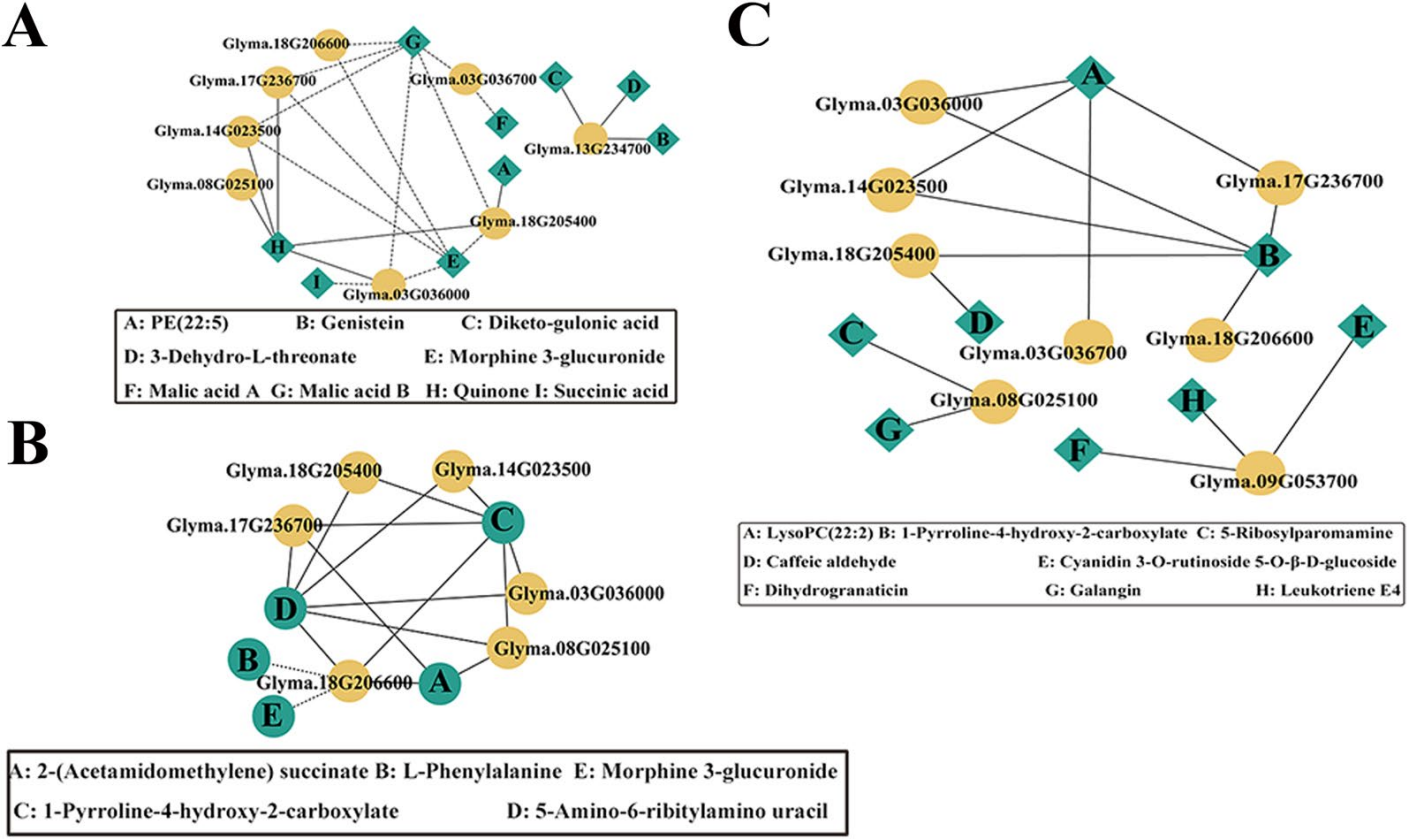
QTN-by-environment detection model and network analysis of candidate DEGs and DAMs in the three comparison groups. **A** FHUFA vs. FLUFA, **B** THUFA vs. TLUFA, and **C** HUFA vs. LUFA. Yellow circles represent genes. Green squares represent metabolites. The solid line represents a positive correlation, while the dashed line represents a negative correlation

In the multiple-environment QEI model, a co-expression network of nine candidate genes and DAMs was constructed for three comparison groups. In the FHUFA vs. FLUFA network, the 22 subnetworks were significantly correlated (|*r*|> 0.5,* p* < 0.05). Quinone was positively associated with *Glyma.08G025100* (*r* > 0.88, *p* < 2.14E−07) and *Glyma.17G236700* (*r* > 0.53, *p* < 0.01) (Fig. 6A). In the THUFA vs. TLUFA network, *Glyma.18G205400* was positively associated with 5-amino-6-ribitylamino uracil (*r* > 0.90, *p* < 9.27E−16) and 1-Pyrroline-4-hydroxy-2-carboxylate (*r* > 0.92, *p* < 2.96E−17). *Glyma.17G236700* was significantly associated with 2-(acetamidomethylene) succinate (*r* > 0.51,* p* < 0.0007), 1-pyrroline-4-hydroxy-2-carboxylate (*r* > 0.69, *p* < 6.09E−07), and 5-Amino-6-ribitylamino uracil (*r* > 0.69, *p* < 6.85E−07) (Fig. 6B). In the HUFA vs. LUFA network, *Glyma.17G236700* was significantly associated with LysoPC (22:2(13Z,16Z)) (*r* > 0.64, *p* < 3.04E−08) and 1-pyrroline-4-hydroxy-2-carboxylate B (*r* > 0.649, *p* < 2.01E−08) (Fig. 6C).

### Gene-based association and haplotype analysis of candidate genes

**Fig. 7:**
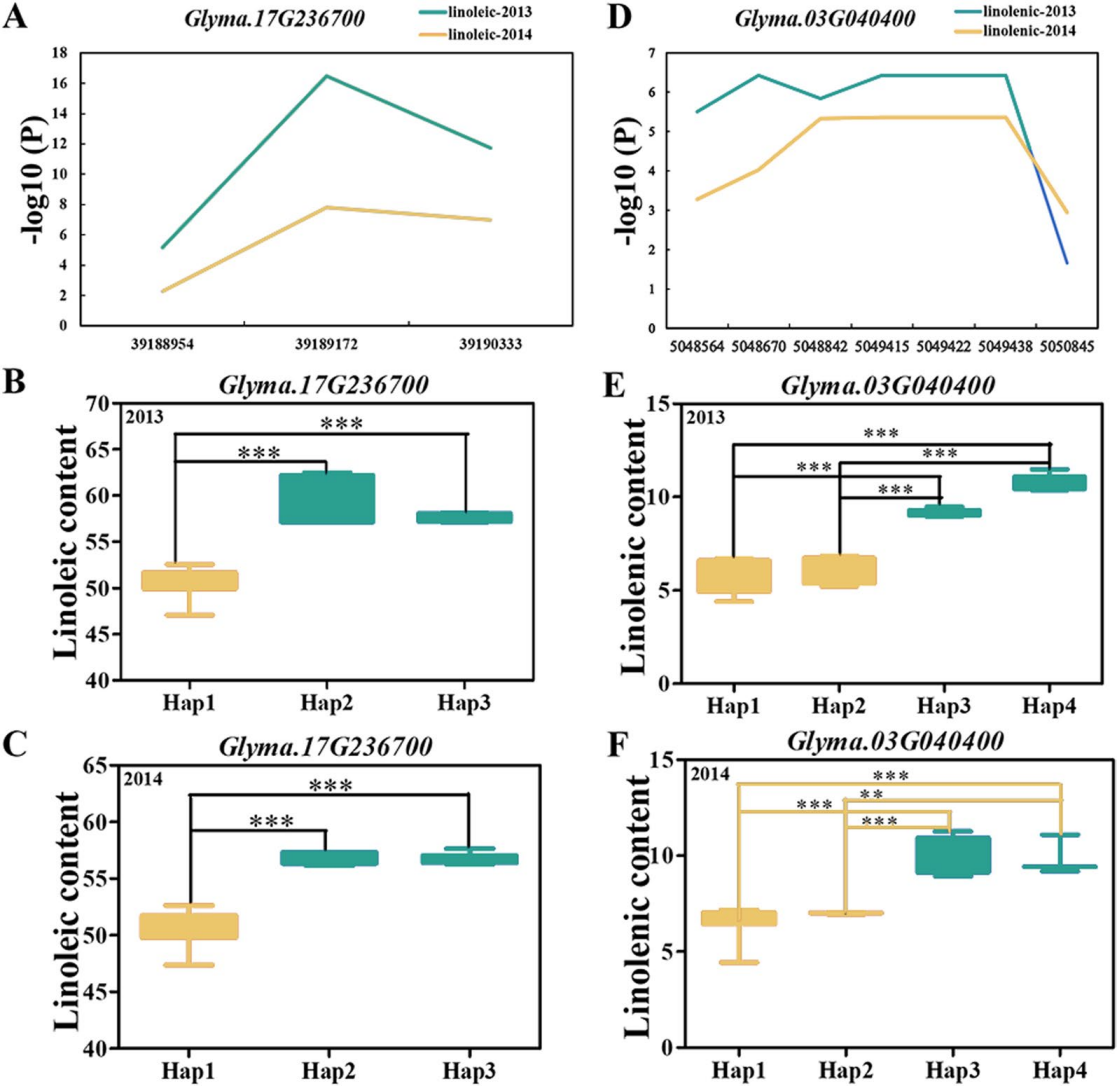
Gene-based association analysis and haplotype analysis. **A** Gene-based association analysis of *Glyma.17G236700* related to linolenic content. **B**, **C** The relationship between haplotypes and linoleic content analysis of *Glyma.17G236700* in 2013 and 2014, respectively. **D** Gene-based association analysis of *Glyma.03G040400* related to linoleic content. **E**, **F** The relationship between haplotypes and linolenic content analysis of *Glyma.03G040400* in 2013 and 2014, respectively. * and ** indicate significance at *p* < 0.05 and *p* < 0.01, respectively

To further determine the relationship between candidate genes and traits, the SNPs of the candidate genes were applied for the gene-based association and haplotype analysis of the candidate genes. According to the results of candidate gene screening based on gene expression data from qRT-PCR and transcriptomics, *Glyma.03G040400* and *Glyma.17G236700*, as the candidate genes of QTNs and overlapping SNPs of QTNs and QEIs, were studied to understand the gene variations affecting soybean unsaturated FAs and to further determine beneficial haplotypes. Three SNPs were found in the promoter and CDS regions of *Glyma.03G040400* (Additional file 1: Table S4). SNP markers 39,188,954, 39,189,172, and 39,190,333 showed an association with LLA (Fig. 7A, Additional file 1: Table S4). Among the three haplotypes of *Glyma.03G040400*, Hap 3 and Hap 2 had a significantly higher LNA content than Hap 1 in 2013 and 2014 (Fig. 7B and C).

For candidate gene *Glyma.17G236700*, seven SNPs were found in the promoter and CDS region. Of these, SNP markers 5,048,564, 5,048,670, 5,048,842, 5,049,415, 5,049,422, 5,049,438, and 5,050,845 were significantly associated with LLA content in 2013 and 2014 (− log10(P) ≥ 2) (Additional file 1: Table S4, Fig. 7D). Four haplotypes of *Glyma.17G236700* were defined by the seven SNPs (Fig. 7E and F). Among the four haplotypes, Hap 3 and Hap 4 had a significantly higher LNA content (2013 and 2014) than Hap 1 and Hap 2.

## Discussion

Soybean is an important oil crop. However, different proportions of FAs may play an important role in soybean oil. Therefore, it is of great significance to improve the content and quality of soybean oil. The single locus method has been widely used to detect genetic variation in crops, including GLM and MLM [23, 24]. However, single-locus GWAS methods generally need Bonferroni correction and can be affected by a polygenic background. In this study, 194 soybean accessions were analyzed using the 3VmrMLM method (Additional file 1: Figure S1, Table 2). We identified 12, 10, and eight significant/suggested SNPs for OA, 17, 10, and seven significant/suggested SNPs for LA, and 10, eight, and 12 significant/suggested SNPs for LLA in 2013, 2014, and 2015, respectively (Table 2). In addition, we compared 3VmrMLM with a single-locus MLM method by Zhao et al. We detected 63 SNPs using the MLM method. Hence, the 3VmrMLM method detected more significant SNPs than the MLM method. Among these SNPs, four SNPs were found using the MLM and 3VmrMLM methods simultaneously, including rs4953186 rs52833743, rs35024325, and rs6481810, and the discovery of rs35024325 and rs6481810 SNPs has been reported [2, 22].

Environmental changes have an important impact on the quality and yield of crops; analysis of multiple environments can increase the detection capability of SNPs. In this study, six, five, and eight QEIs were found for OA, LA, and LLA, respectively (Fig. 2, Table 3). Among these SNPs, five have been reported [22, 25, 26]. A total of 1246 genes around the significant/suggested QTNs were predicted in this study; of them, 40 genes were involved in lipid synthesis (Additional file 1: Table S1). For example, the MYB transcription factor has been reported to affect oil accumulation [27]. The *OsLTP* gene is involved in the transport of lipid molecules in rice [28]. In this study, *Glyma.03G040400* (*GmLTP1*), located on chromosome 3, was significantly related to LNA using the GLM method based on gene-based association (Additional file 1: Table S4). In addition, the *GmLTP1* gene was a beneficial haplotype (Fig. 7).

A total of 301 genes around the significant/suggested QEIs were detected in this study (Additional file 1: Table S2). Three significant QEIs, namely rs4633292, rs39216169, and rs14264702, overlapped with significant single-environment QTNs. Among the overlapping SNPs, genes related to FA synthesis and seed development, such as ACBP and FTSH, were identified. ACBPs can play an important role in maintaining lipid homeostasis [29]. In addition, we found that the *Glyma.17G236700* (ACBP) gene had a beneficial haplotype (Fig. 7).

## Conclusion

The 3VmrMLM method was more comprehensive for GWAS. This method overcame the huge computational burden of traditional models. In this study, 94 QTNs and 19 QEIs were identified. Five major candidate genes were found. The gene expression data from different soybean tissues and transcriptome data were used to identify *Glyma.03G040400* and *Glyma.17G236700* as key candidate genes around the SNPs. The beneficial haplotypes of *Glyma.03G040400* and *Glyma.17G236700* may be helpful for further application in soybean breeding.

## Methods

### Plant materials, field trials, and phenotypic evaluation

An association panel of 194 soybean germplasm resources was planted at Harbin (162.41° E, 45.45° N) in 2013, 2014, and 2015. Field trials were conducted using single-row plots (2 m long and 0.65 m between rows) and a randomized complete block design with three replicates per experimental site. The unsaturated FA content of each sample was determined using gas chromatography (GC-14C, Shimadzu Company, Japan), according to our previous method [30]. The OA, LLA, and LNA content were applied in single-environment (QTN) and multi-environment (QEI) analyses.

### Genotypic data

**Fig. 8:**
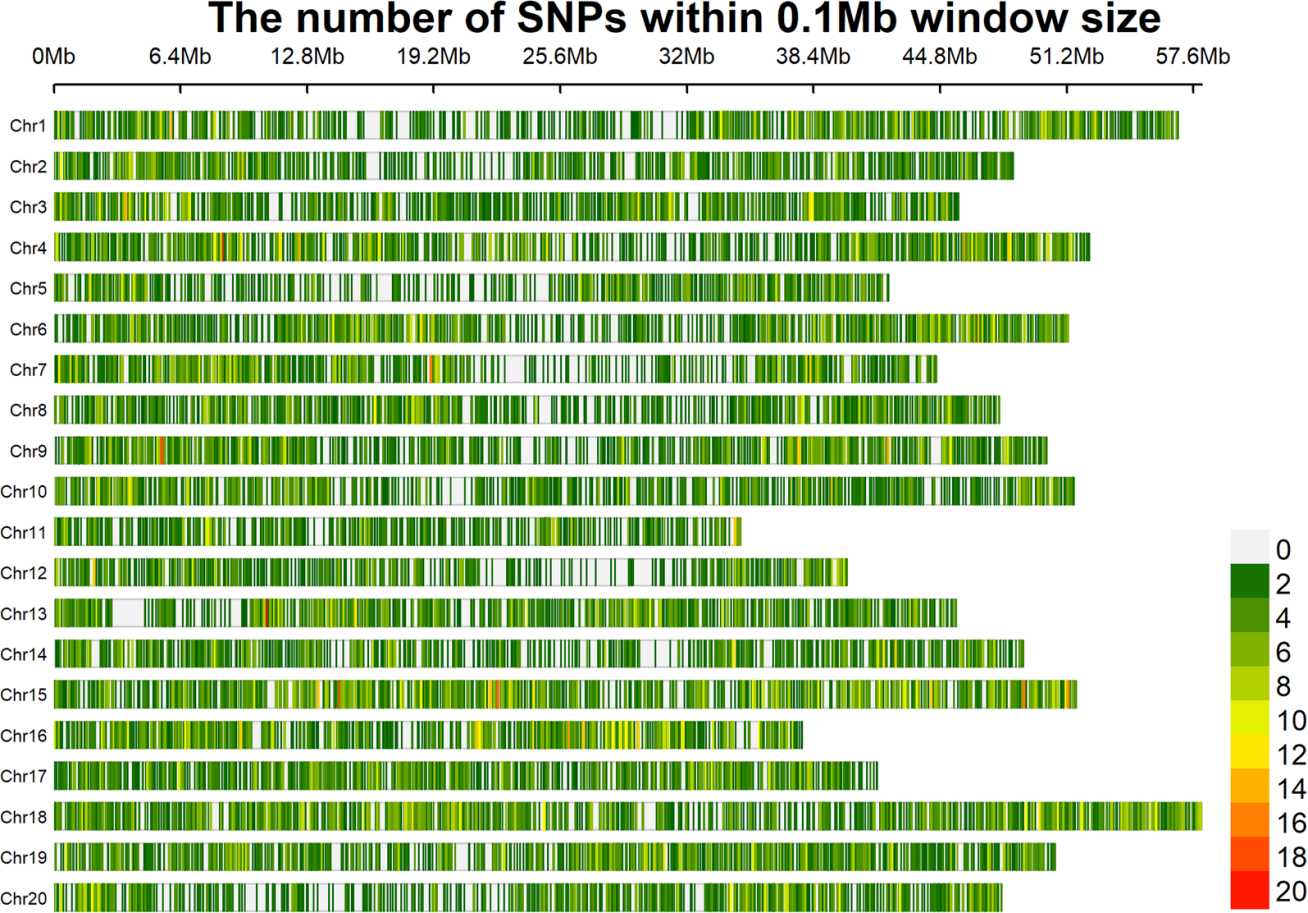
Density distribution of single nucleotide polymorphisms (SNPs)

A genotypic dataset consisting of 36,981 SNPs from 194 soybean germplasm resources was generated by Specific-Locus Amplified Fragment Sequencing (SLAF-seq), which was reported in Han et al. and Zhao et al. [2, 31]. The 36,981 SNPs were distributed on 20 soybean chromosomes, with minor allele frequencies > 0.04 and missing data of < 10% (Fig. 8).

### GWAS

The 36,981 SNPs and unsaturated FA content of 194 soybean accessions were used for association analysis via the 3VmrMLM method in 3VmrMLM software [21]. QTNs for OA, LLA, and LNA content were calculated from a single environment (3 years of phenotypic data from 2013, 2014, and 2015). QEIs for OA, LLA, and LNA content were calculated using a joint analysis of multiple environments. The threshold of significance for QTNs and QEIs was set at *p* = 0.05 and LOD score ≥ 3.0.

### Differential expression analysis based on RNA-seq

At the R6 stage, 30 soybean varieties with a high content of three unsaturated FAs and a low content of three unsaturated FAs were collected for RNA sequencing (RNA-seq) with two biological replicates. Total RNA was extracted using TRIzol reagent (Invitrogen, Carlsbad, CA, USA). The cDNA sequencing libraries of 18 RNA samples were constructed and sequenced, and RNA-seq data were generated using the Illumina platform. Differentially expressed genes (DEGs) were identified using the edgeR package in R software [32]. The significance level was set as follows: |log2(fold change)|≥ 1.

### Identification of candidate genes

The 100-kb flanking region of each identified QTN and QEI was defined to search for candidate genes according to linkage disequilibrium decay analysis, as described in Zhao et al. [2]. Candidate genes for unsaturated FAs were extracted in the following steps. According to previous reports, known genes related to FA content in *Arabidopsis* were considered references to screen their homologous genes in the soybean genome. The new candidate genes were identified using DEGs for unsaturated FAs.

### Metabolite profiling

The non-targeted metabolome was completed by Bioacme Biotechnology Co., Ltd. (Wuhan, China). Briefly, a 100 mg soybean sample was loaded into a 2-mL centrifuge tube, and 300 μL 75% methanol/water was added. The tubes were centrifuged at 12,000 rpm for 15 min at 4 °C. Metabolites were screened and identified using the Metlin database. The differential metabolites were calculated using an orthogonal partial least squares-discriminant analysis (OPLS-DA) model, with a variable importance in the projection (VIP) score of ≥ 1 and a |log2 (fold change)| of ≥ 1.

### Haplotype analysis and gene-based association analysis of candidate genes

The SNP variation of candidate genes was analyzed based on genome sequencing data. These SNPs were located at the full length of the gene, including exons, intronic regions, and upstream and downstream of the gene. Therefore, in this study, phenotypic data, including high and low total unsaturated FA content, from 50 soybean germplasm resources were used over 3 years to conduct an association analysis. A general linear model (GLM) was used to further determine the association between the SNP variation of candidate genes and unsaturated FA content using TASSEL software [33]. Significant SNP variation in candidate genes was considered when the* P* value was less than 0.01.

### Quantitative real-time PCR (qRT-PCR)

Soybean seeds with high and low unsaturated FA content were collected at the R6 stage. Total RNA was extracted using the TRIzol method, and cDNA was generated using the ReverTra Ace qPCR RT Master Mix (TOYOBO, Osaka, Japan). Real-time quantitative PCR (qRT-PCR) was performed on an ABI 7500 fast real-time PCR platform with SYBR Green (TOYOBO, Osaka, Japan). *GmACTIN4* was used as an internal control, and the primer sequences for candidate genes are listed in Additional file 1: Table S5. The L-13 soybean seed samples were used as a calibrator. The results of qRT-PCR were calculated using the 2^−ΔΔCT^ method [34].

### Co-expression analysis

The correlation coefficient was calculated between candidate genes and DAM metabolites, and a Pearson correlation cutoff value of 0.5 was generated. Data were visualized using the Cytoscape package [35].

### Statistical analysis

Statistical significance was evaluated using Student’s t-test performed with SPSS 22.0 software (IBM Corp., Armonk, NY, USA). “*” and “**” represent a significance level of* p* < 0.05 and *p* < 0.01, respectively. The mean and standard deviation (mean ± SD) were calculated using the data from three biological replicates.

### Supplementary Information

**Additional file 1: Table S1.** QTNs identified for unsaturated fatty acids content using the QTN detection model in 3VmrMLM. **Table S2.** QEIs identified for unsaturated fatty acids content three environments detected using the QTN-by-environment detection model in 3VmrMLM. **Table S3.** Unsaturated fatty acids content of the 30 soybean varieties. **Table S4.** The association between SNP in *Glyma.17G23670* and *Glyma.03G040400* gene and soybean unsaturated fatty acids content based on 50 soybean germplasms. **Table S5.** Primers used for qRT-PCR. **Figure S1.** Manhattan plots of the single-environment analysis for the oleic, linoleic and linolenic traits in 2013, 2014 and 2015 of soybean. **Figure S2.** A and B KEGG pathway annotation around QTN and QEI candidate genes, respectively. **Figure S3.** Candidate genes are identified in the transcriptome and (A): QTN detection model, (B): QTN-by-environment detection model. **Figure S4.** Analysis of candidate genes by qRT-PCR. **Figure S5.** Differential accumulation of metabolite in the three comparison groups.

## Data Availability

All data generated or analyzed during this study are included in this published article and its additional files.
