# Supplementary figures and images for: Multi-omics analysis reveals novel loci and a candidate regulatory gene of unsaturated fatty acids in soybean (Glycine max (L.) Merr)
Source: Biotechnol Biofuels Bioprod. 2024 Mar 16;17:43. doi: 10.1186/s13068-024-02489-2 (PMC10944593; doi:10.1186/s13068-024-02489-2)

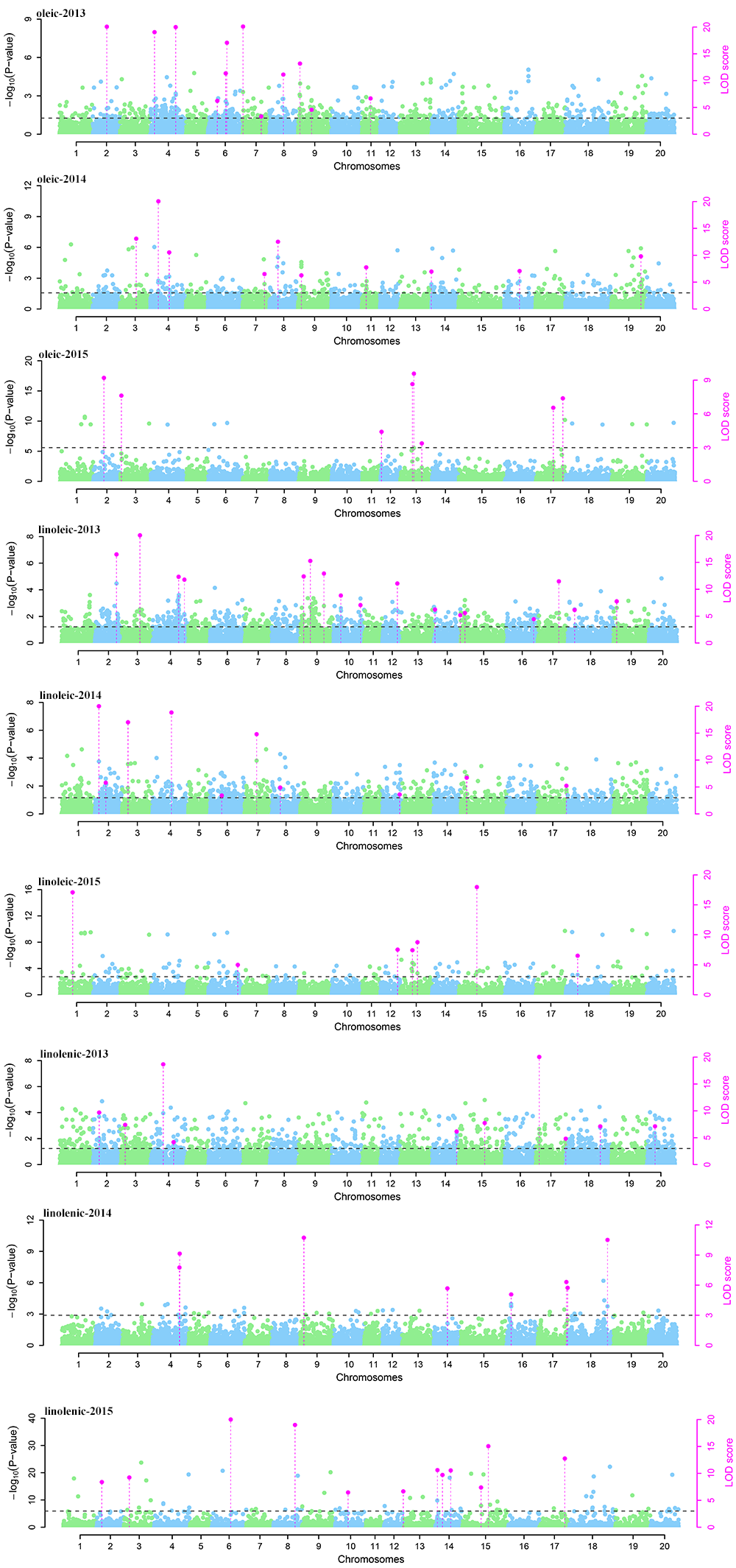

Supplement: Supplementary file 1 — Additional file 1: Table S1. QTNs identified for unsaturated fatty acids content using the QTN detection model in 3VmrMLM. Table S2. QEIs identified for unsaturated fatty acids content three environments detected using the QTN-by-environment detection model in 3VmrMLM. Table S3. Unsaturated fatty acids content of the 30 soybean varieties. Table S4. The association between SNP in Glyma.17G23670 and Glyma.03G040400 gene and soybean unsaturated fatty acids content based on 50 soybean germplasms. Table S5. Primers used for qRT-PCR. Figure S1. Manhattan plots of the single-environment analysis for the oleic, linoleic and linolenic traits in 2013, 2014 and 2015 of soybean. Figure S2. A and B KEGG pathway annotation around QTN and QEI candidate genes, respectively. Figure S3. Candidate genes are identified in the transcriptome and (A): QTN detection model, (B): QTN-by-environment detection model. Figure S4. Analysis of candidate genes by qRT-PCR. Figure S5. Differential accumulation of metabolite in the three comparison groups. [file 13068_2024_2489_MOESM1_ESM.zip › Supplementary material/FigureS1.tif]

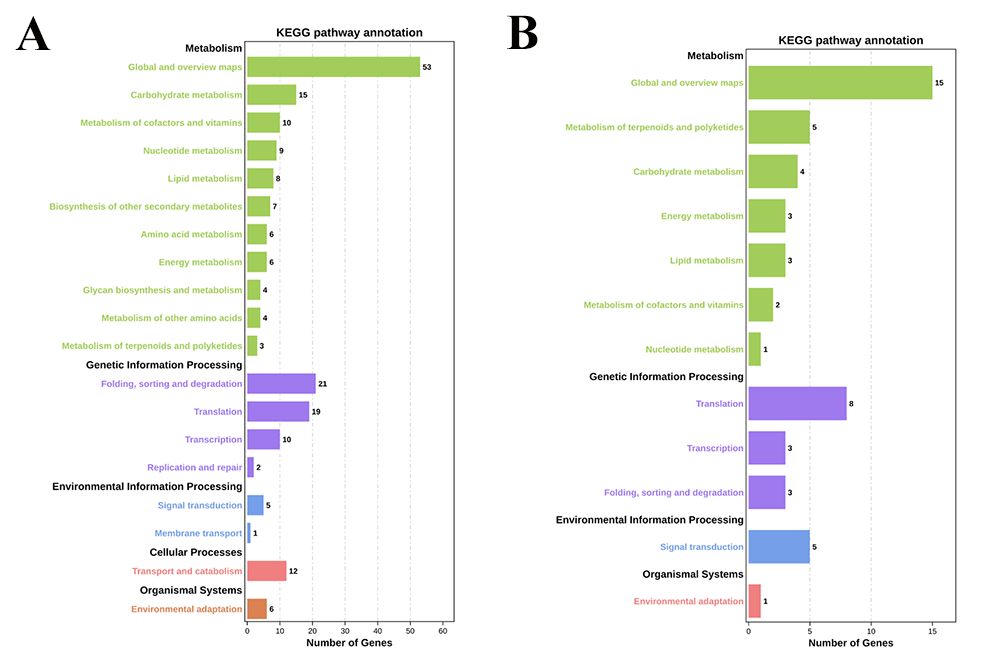

Supplement: Supplementary file 1 — Additional file 1: Table S1. QTNs identified for unsaturated fatty acids content using the QTN detection model in 3VmrMLM. Table S2. QEIs identified for unsaturated fatty acids content three environments detected using the QTN-by-environment detection model in 3VmrMLM. Table S3. Unsaturated fatty acids content of the 30 soybean varieties. Table S4. The association between SNP in Glyma.17G23670 and Glyma.03G040400 gene and soybean unsaturated fatty acids content based on 50 soybean germplasms. Table S5. Primers used for qRT-PCR. Figure S1. Manhattan plots of the single-environment analysis for the oleic, linoleic and linolenic traits in 2013, 2014 and 2015 of soybean. Figure S2. A and B KEGG pathway annotation around QTN and QEI candidate genes, respectively. Figure S3. Candidate genes are identified in the transcriptome and (A): QTN detection model, (B): QTN-by-environment detection model. Figure S4. Analysis of candidate genes by qRT-PCR. Figure S5. Differential accumulation of metabolite in the three comparison groups. [file 13068_2024_2489_MOESM1_ESM.zip › Supplementary material/FigureS2.tif]

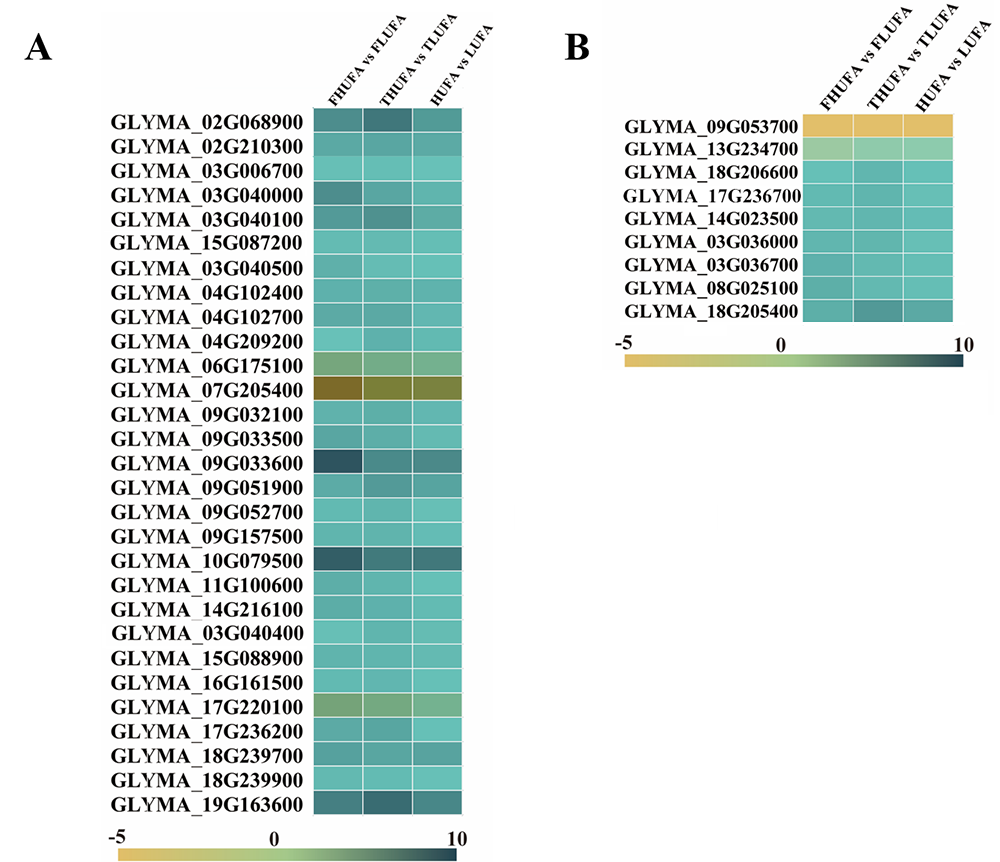

Supplement: Supplementary file 1 — Additional file 1: Table S1. QTNs identified for unsaturated fatty acids content using the QTN detection model in 3VmrMLM. Table S2. QEIs identified for unsaturated fatty acids content three environments detected using the QTN-by-environment detection model in 3VmrMLM. Table S3. Unsaturated fatty acids content of the 30 soybean varieties. Table S4. The association between SNP in Glyma.17G23670 and Glyma.03G040400 gene and soybean unsaturated fatty acids content based on 50 soybean germplasms. Table S5. Primers used for qRT-PCR. Figure S1. Manhattan plots of the single-environment analysis for the oleic, linoleic and linolenic traits in 2013, 2014 and 2015 of soybean. Figure S2. A and B KEGG pathway annotation around QTN and QEI candidate genes, respectively. Figure S3. Candidate genes are identified in the transcriptome and (A): QTN detection model, (B): QTN-by-environment detection model. Figure S4. Analysis of candidate genes by qRT-PCR. Figure S5. Differential accumulation of metabolite in the three comparison groups. [file 13068_2024_2489_MOESM1_ESM.zip › Supplementary material/FigureS3.tif]

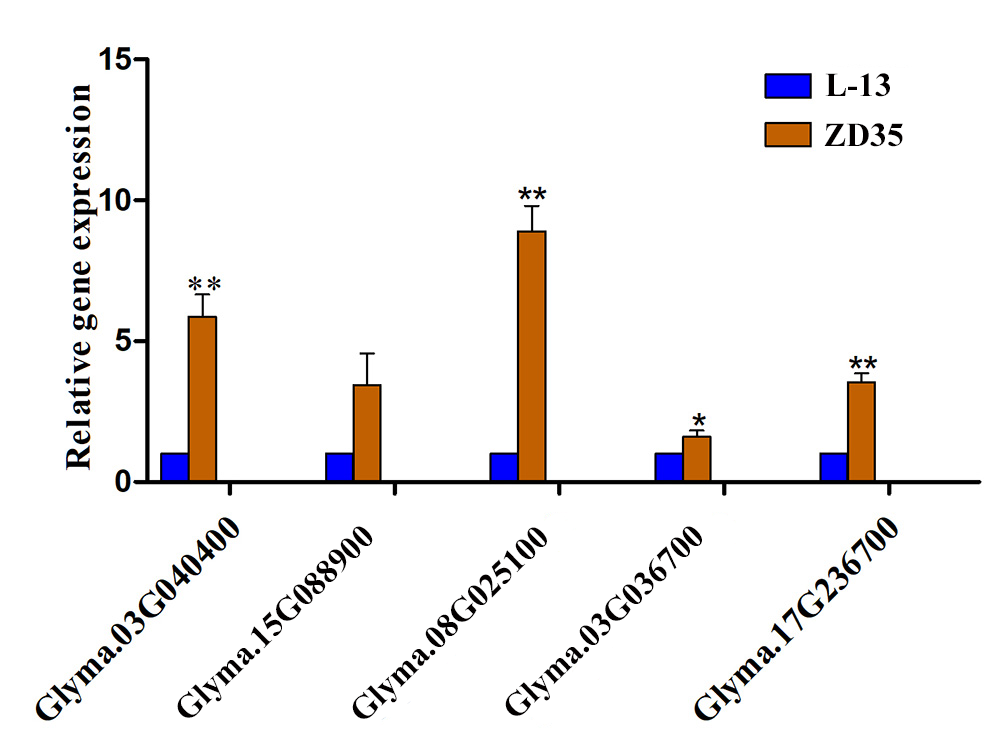

Supplement: Supplementary file 1 — Additional file 1: Table S1. QTNs identified for unsaturated fatty acids content using the QTN detection model in 3VmrMLM. Table S2. QEIs identified for unsaturated fatty acids content three environments detected using the QTN-by-environment detection model in 3VmrMLM. Table S3. Unsaturated fatty acids content of the 30 soybean varieties. Table S4. The association between SNP in Glyma.17G23670 and Glyma.03G040400 gene and soybean unsaturated fatty acids content based on 50 soybean germplasms. Table S5. Primers used for qRT-PCR. Figure S1. Manhattan plots of the single-environment analysis for the oleic, linoleic and linolenic traits in 2013, 2014 and 2015 of soybean. Figure S2. A and B KEGG pathway annotation around QTN and QEI candidate genes, respectively. Figure S3. Candidate genes are identified in the transcriptome and (A): QTN detection model, (B): QTN-by-environment detection model. Figure S4. Analysis of candidate genes by qRT-PCR. Figure S5. Differential accumulation of metabolite in the three comparison groups. [file 13068_2024_2489_MOESM1_ESM.zip › Supplementary material/FigureS4.tif]

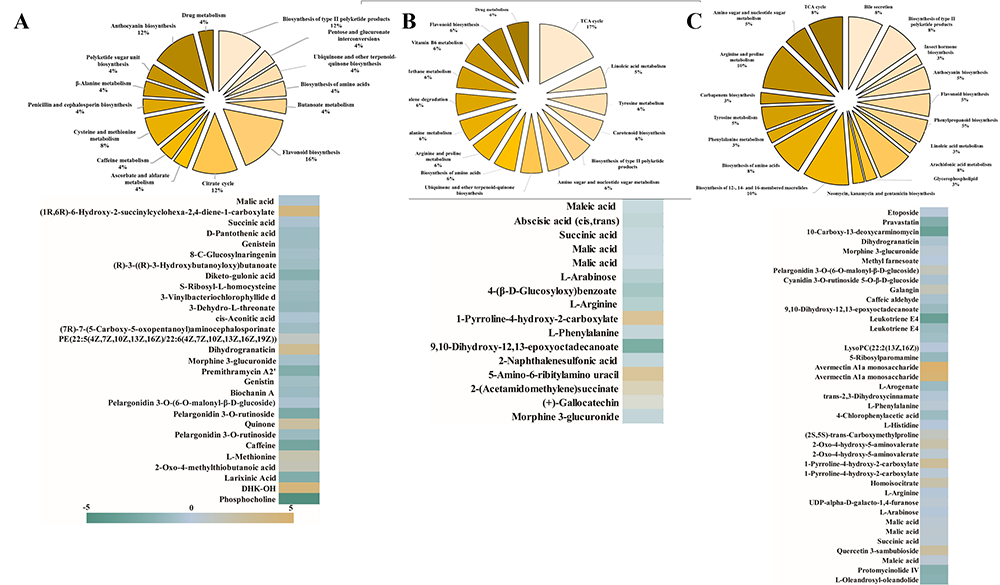

Supplement: Supplementary file 1 — Additional file 1: Table S1. QTNs identified for unsaturated fatty acids content using the QTN detection model in 3VmrMLM. Table S2. QEIs identified for unsaturated fatty acids content three environments detected using the QTN-by-environment detection model in 3VmrMLM. Table S3. Unsaturated fatty acids content of the 30 soybean varieties. Table S4. The association between SNP in Glyma.17G23670 and Glyma.03G040400 gene and soybean unsaturated fatty acids content based on 50 soybean germplasms. Table S5. Primers used for qRT-PCR. Figure S1. Manhattan plots of the single-environment analysis for the oleic, linoleic and linolenic traits in 2013, 2014 and 2015 of soybean. Figure S2. A and B KEGG pathway annotation around QTN and QEI candidate genes, respectively. Figure S3. Candidate genes are identified in the transcriptome and (A): QTN detection model, (B): QTN-by-environment detection model. Figure S4. Analysis of candidate genes by qRT-PCR. Figure S5. Differential accumulation of metabolite in the three comparison groups. [file 13068_2024_2489_MOESM1_ESM.zip › Supplementary material/FigureS5.tif]
